# Study on pathological and clinical characteristics of chronic HBV infected patients with HBsAg positive, HBV DNA negative, HBeAg negative

**DOI:** 10.3389/fimmu.2022.1113070

**Published:** 2023-01-05

**Authors:** Zhan Zeng, Ruyu Liu, Weihua Cao, Liu Yang, Yanjie Lin, Xiaoyue Bi, Tingting Jiang, Wen Deng, Shiyu Wang, Huihui Lu, Fangfang Sun, Ge Shen, Min Chang, Yao Lu, Shuling Wu, Hongxiao Hao, Mengjiao Xu, Xiaoxue Chen, Leiping Hu, Lu Zhang, Gang Wan, Yao Xie, Minghui Li

**Affiliations:** ^1^ Department of Hepatology Division 2, Beijing Ditan Hospital, Capital Medical University, Beijing, China; ^2^ Department of Infectious Diseases, Peking University First Hospital, Beijing, China; ^3^ Department of Hepatology Division 2, Peking University Ditan Teaching Hospital, Beijing, China; ^4^ Department of Biostatistics, Beijing Ditan Hospital, Capital Medical University, Beijing, China

**Keywords:** HBV DNA, HBeAg, chronic HBV-infection, clinical indicators, liver histopathology

## Abstract

**Aims:**

Study of clinical characteristics of hepatitis B virus deoxyribonucleic acid (HBV DNA)-negative, hepatitis B surface antigen (HBsAg)-positive, hepatitis B e antigen (HBeAg)-negative patients based on liver histopathology.

**Methods:**

We retrospectively enrolled patients with chronic HBV infection diagnosis at Beijing Ditan Hospital from May 2008 to November 2020. To study the differences between patients with significant hepatic histopathology and those without significant hepatic histopathology. And to study the independent factors of significant hepatic histopathology.

**Results:**

85 HBV DNA-negative and HBeAg-negative patients were 37.90 ± 10.30 years old, 23.50% of patients with grade of inflammation (G) >1, 35.30% of patients with liver fibrosis stage (S) >1, 44.70% patients were diagnosed with significant hepatic histopathology. Compared to the no significant hepatic histopathology group, another group had older age (41.70 ± 10.70 vs 34.80 ± 8.87 years, t=-3.28, *P*=0.002), higher total bilirubin (TBIL) [14.9(10.3, 22.4) vs 11(8.9, 14.4) μmol/L, z=-2.26, *P*=0.024], lower cholinesterase (CHE) (t=-2.86, *P*=0.005, 7388.00 ± 2156.00 vs 8988.00 ± 2823.00 U/L) and lower platelet (PLT) (t=2.75, *P*=0.007, 157.00 ± 61.40 vs 194.00 ± 61.00 10^9/L). Abnormal ALT patients are more likely to have significant hepatic histopathology (z=5.44, *P*=0.020, 66.70% vs 337.50%). G had significant correlation with CHE (*P*=0.008, r=-0.23), alanine aminotransferase (ALT) (*P*=0.041, r=0.18), aspartate aminotransferase (AST) (*P*=0.001, r=0.29). S had significant correlation with TBIL (*P* = 0.008, r = 0.23), age (*P* < 0.001, r = 0.32), international normalized ratio (INR) (*P* = 0.04, r = 0.23), CHE (*P* < 0.001, r = -0.30), PLT (*P* < 0.001, r = -0.40) and prothrombin time activity (PTA) (*P* = 0.046, r = -0.22). Multivariate logistic analysis indicated only age (95%CI=1.014~1.130, OR=1.069, *P*=0.013) was an impact factor for significant hepatic histopathology. The cutoff point of age was 34.30 years.

**Conclusions:**

A large proportion of chronic HBV infection patients with HBeAg-negative and HBV DNA-negative still have chronic hepatitis. Age is an independent factor for significant hepatic histopathology

## Introduction

As reported in the latest (2017) world health organization (WHO) global hepatitis report, in 2015, there were approximately 257 million people infected hepatitis B virus (HBV) worldwide (1). Chronic HBV infection has 5 periods of natural history, which contain hepatitis B e antigen (HBeAg)-positive chronic HBV infection, HBeAg-negative chronic HBV infection, HBeAg-positive chronic hepatitis B, HBeAg-negative chronic hepatitis B, hepatitis B surface antigen (HBsAg)-negative phase (2), and each period has its characteristics.

HBeAg-negative chronic HBV infection is characterized by HBeAb-positive, normal alanine aminotransferase (ALT), and low HBV DNA load (3). In the past, this period was termed as “inactive carriers (IC)”, but as understanding of HBV disease history and immune mechanisms has improved, scholars have replaced the term IC with HBeAg-negative chronic HBV infection (4). Although prognosis for patients in this period is said to be good, some patients may still experience fluctuations in HBV DNA and/or ALT. These fluctuations may then lead to disease progression such as liver fibrosis, liver cirrhosis or even liver cancer (5, 6). Previously, many scholars have studied the clinical features and prognosis of this stage. One limitation of those studies is that they did not use the high-sensitivity HBV DNA quantification assay. Our research was conducted to study the histopathological characteristics and clinical indicators of HBeAg-negative, HBV DNA-negative patients using a high-sensitivity assay in combination with liver biopsy.

## Materials and methods

### Patients

Chronic HBV-infected patients who underwent liver biopsies were obtained from Beijing Ditan Hospital affiliated to Capital Medical University between May 2008 and November 2020. Information on antiviral therapy, demographic data, HBV serological test and HBV DNA load results, renal function, liver function, blood routine examination and blood coagulation function were collected.

Admission criteria: 1) HBsAg-positive for over 6 months; 2) 18 to 65 years old; 3) no HBV treatment received; 4) complete viral load and viral serology results, coagulation, liver function, blood routine within 2 weeks after or before biopsy; 5) have clear diagnostic results of liver biopsy.

Exclusion criteria: 1) infected with other viruses (e.g., HIV, cytomegalovirus, etc.); 2) Co-infection with other viral hepatitis (such as hepatitis C, A, D and E), non-alcoholic fatty liver disease, alcoholic liver disease, autoimmune liver disease, drug-induced liver injury, cirrhosis, hepatoma; 3) patients with cardiac or renal dysfunction; 4) patients who have not obtained a clear result of inflammatory grade or liver fibrosis stage.

### Study parameters

ALT, AST, HBsAg, HBeAg, HBV DNA, albumin (ALB), international normalized ratio (INR), total bilirubin (TBIL), cholinesterase (CHE), prothrombin time activity (PTA), platelet (PLT).

Liver pathology results included liver fibrosis stage S0-S4, and inflammatory grade G0-G4.

In our study, S>1 indicates significant liver fibrosis, 0<S ≤ 1 indicates slight liver fibrosis, S0 indicates no liver fibrosis. G>1 indicates significant liver inflammation, and G ≤ 1 indicates no significant liver inflammation. No significant hepatic histopathology group’s patients meet G ≤ 1 and S ≤ 1, and significant hepatic histopathology group’s patients meet G>1 and/or S>1.

### Study contents

To study the correlation factors associated with the pathological grading of liver biopsies in HBeAg-negative patients with chronic HBV infection and to analyze the characteristics of their inflammatory grading and fibrosis staging. To compare the differences in clinical indicators between the two groups of patients. To study the risk factors (or protective factors) for significant liver histopathology and calculate the cutoff values of the relevant factors.

### Statistical analysis

Use the mean ± standard deviation or quartiles to describe enumeration data and percentages were used to describe categorical data. Categorical data were compared using chi-square test. Student’s t-test was used to compare normally distributed data. Using Mann-Whitney test to compare non-normally distributed data. Risk factors (or protective factors) were studied using binary logistic regression analysis, and cutoff values for relevant risk factors (or protective factors) were calculated from youden’s index and receiver operating curve (ROC). The software involving figures making was Microsoft Excel 2019 and Graphpad Prism 8.0. Statistical analysis was performed in IBM SPSS 25.0. *P*<0.05 indicates statistical significance.

## Results

### Patients

**Figure 1:**
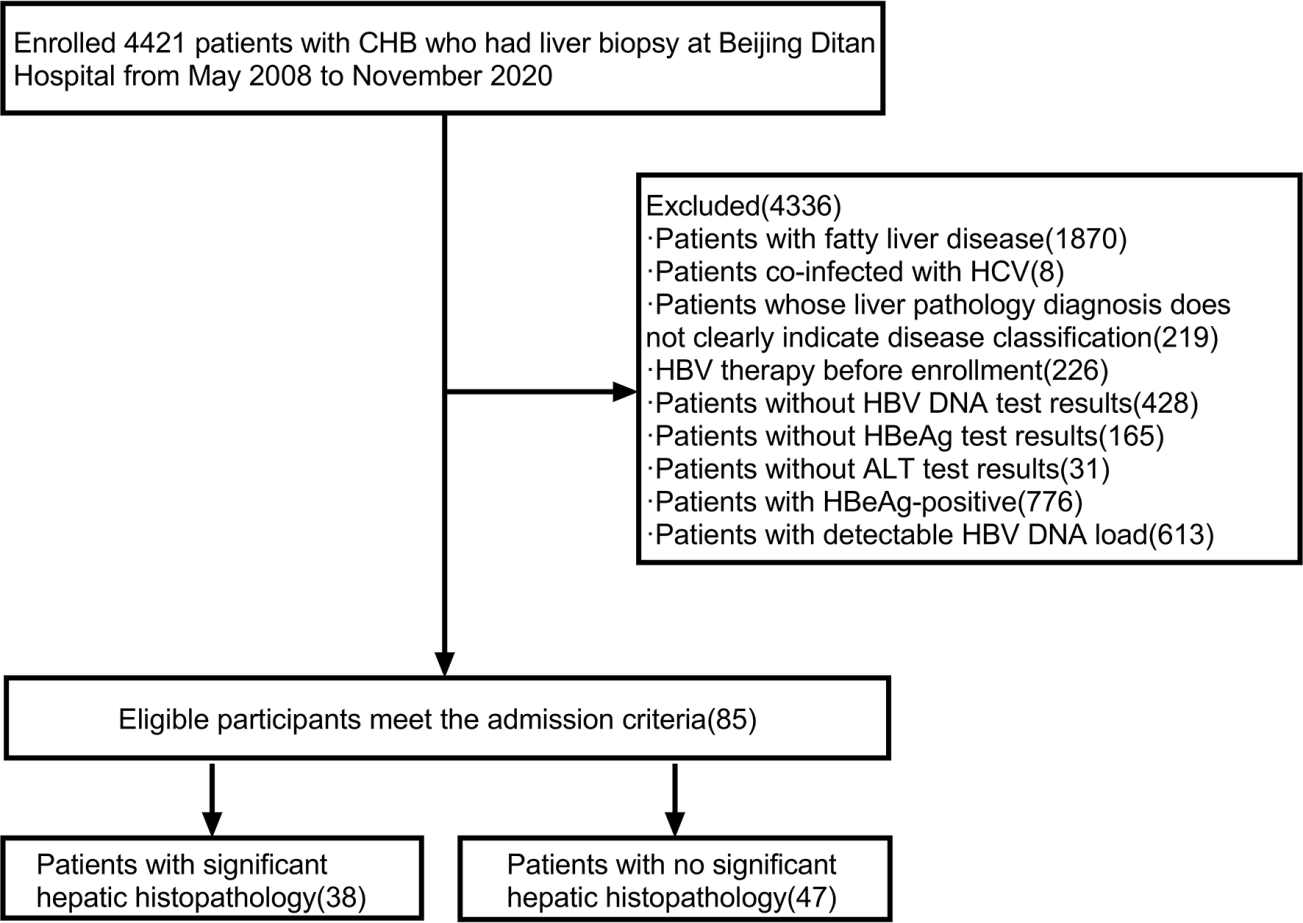
Flow chart for screening research patients. Abbreviations: ALT, alanine aminotransferase; CHB, chronic hepatitis B; HBV DNA, hepatitis B virus deoxyribonucleic acid; HBV, hepatitis B virus; HCV, hepatitis C virus; HBeAg, hepatitis B e antigen.

A total of 4421 HBV infected people had liver puncture biopsies. After collecting HBV serologic indicators, HBV DNA load, demographic data, clinical biochemical indicators, and blood routines. We performed screening and excluded 8 patients with hepatitis C, 1870 patients with fatty liver, 219 patients whose liver pathology diagnosis did not clearly show disease grade, 226 patients who received HBV treatment, 165 patients without HBeAg results, 428 patients without HBV DNA load results, 31 patients without ALT results, 613 patients with a detectable HBV DNA load, 776 HBeAg-positive patients. Finally, a total of 85 HBeAg-negative, HBV DNA-negative patients (HBeAg-negative, HBV DNA load below the lower limit of detection) were included in this study. (**Figure 1**)

In this group of patients, 57.60% were male, age (37.90 ± 10.30) years old, significant hepatic histopathology was observed in 44.70% of patients. Compared with no significant hepatic histopathology group, the other group had older age (41.70 ± 10.70 vs 34.80 ± 8.87 years, t=-3.28, *P*=0.002), higher TBIL levels [14.9(10.3, 22.4) vs 11(8.9, 14.4) μmol/L, z=-2.26, *P*=0.024], lower CHE levels (7388.00 ± 2156.00 vs 8988.00 ± 2823.00 U/L, t=-2.86, *P*=0.005) and PLT counts (157.00 ± 61.40 vs 194.00 ± 61.00 10^9/L, t=2.75, *P*=0.007). (**Table 1**)

**Table 1:** Characteristics and comparison of clinical indicators in HBeAg-negative, HBV DNA-negative patients.

Characteristics	Total(N=85)	No significant hepatic histopathology (n=47)	Significant hepatic histopathology (n=38)	t/*χ^2^*/*z*	*P*
**Male(%)**	57.60%	55.30%	60.50%	0.23	0.629
**Age(years)**	37.90 ± 10.30	34.80 ± 8.87	41.70 ± 10.70	-3.28	0.002
**HBsAg(log_10_ IU/ml)**	2.01 ± 1.49	2.21 ± 1.59	1.81 ± 1.38	0.98	0.33
**ALT(U/L)**	25.70(17.50, 39.80)	24.00(16.50, 33.50)	30.10(19.60, 54.80)	-1.70	0.09
**AST(U/L)**	22.80(17.80, 33.20)	22.30(17.60, 28.30)	26.30(17.90, 39.50)	-1.94	0.053
**TBIL(μmol/L)**	12.30(9.30, 17.50)	11.00(8.90, 14.40)	14.90(10.30, 22.40)	-2.26	0.024
**ALB(g/L)**	44.90 ± 4.89	45.40 ± 4.01	44.30 ± 5.79	1.04	0.303
**CHE(U/L)**	8255.00 ± 2649.00	8988.00 ± 2823.00	7388.00 ± 2156.00	2.86	0.005
**PLT(10^9^/L)**	177.00 ± 63.60	194.00 ± 61.00	157.00 ± 61.40	2.75	0.007
**PTA(%)**	88.80 ± 14.50	90.90 ± 14.00	86.40 ± 14.90	1.13	0.263
**INR**	1.02 ± 0.12	1.01 ± 0.11	1.03 ± 0.13	-0.88	0.382
Characteristics	Total(N=85)	normal ALT (n=64)	abnormal ALT (n=21)	t/*χ^2^*/*z*	*P*
**Significant hepatic histopathology(%)**	44.70%	37.50%	66.70%	5.44	0.020
**Male(%)**	57.60%	53.10%	71.4%	2.17	0.141
**Age(years)**	37.90 ± 10.30	37.63 ± 10.52	38.62 ± 9.60	-0.38	0.702
**HBsAg(log_10_ IU/ml)**	2.01 ± 1.49	1.94 ± 1.56	2.18 ± 1.33	-0.52	0.608
**ALT(U/L)**	25.70(17.50, 39.80)	21.9(14.9, 29.8)	67.8(56.3, 97.5)	-6.85	<0.001
**AST(U/L)**	22.80(17.80, 33.20)	21.8(17, 24.6)	45.5(35.8, 87.4)	-6.41	<0.001
**TBIL(μmol/L)**	12.30(9.30, 17.50)	11.3(9.15, 17.5)	14.9(12.3, 17.8)	-1.74	0.083
**ALB(g/L)**	44.90 ± 4.89	44.83 ± 4.78	45.28 ± 5.32	-0.36	0.721
**CHE(U/L)**	8255.00 ± 2649.00	8308.97 ± 2754.34	8096.15 ± 2366.06	0.32	0.752
**PLT(10^9^/L)**	177.00 ± 63.60	180.39 ± 63.67	166.49 ± 63.97	0.84	0.406
**PTA(%)**	88.80 ± 14.50	88.31 ± 14.4	90.59 ± 15.42	-0.46	0.648
**INR**	1.02 ± 0.12	1.03 ± 0.12	0.99 ± 0.11	0.90	0.373

ALT, alanine aminotransferase; ALB, albumin; AST, aspartate aminotransferase; TBIL, total bilirubin; HBsAg, hepatitis B surface antigen; HBV DNA, hepatitis B virus deoxyribonucleic acid; CHE, cholinesterase; PTA, prothrombin time activity; PLT, platelet; INR, international normalized ratio; /, or.

Compared with normal ALT group, abnormal ALT group had more patients with significant hepatic histopathology (66.70% vs 337.50%, z=5.44, *P* =0.020). (**Table 1**)

### Liver histopathology

In liver histopathological diagnosis, liver inflammation grading: 76.50% (65 cases) of patients with G ≤ 1, 15.30% (13 cases) of patients with 1<G ≤ 2, and 8.20% (7 cases) of patients with 2<G ≤ 3. Liver fibrosis grading: S0 patients accounted for 1.20% (1 case), S ≤ 1 patients accounted for 63.50% (54 cases), 1<S ≤ 2 patients accounted for 24.70% (21 cases), 2<S ≤ 3 patients accounted for 8.20% (7 cases), and 3<S ≤ 4 patients accounted for 2.40% (2 cases) (**Table 2**).

**Table 2:** Histopathological grade distribution table for liver biopsies of HBeAg-negative and HBV DNA-negative patients.

Classification	Degree of Histopathology	Grade	Number of people (%)
**G**	Insignificant	G0	0
		G ≤ 1	76.50%
	Significant	1<G ≤ 2	15.30%
		2<G ≤ 3	8.20%
		3<G ≤ 4	0
**S**	Insignificant	S0	1.20%
		S ≤ 1	63.50%
	Significant	1<S ≤ 2	24.70%
		2<S ≤ 3	8.20%
		3<S ≤ 4	2.40%

G, inflammation grade; S, liver fibrosis stage.

### Correlation between pathological grading and clinical indicators

The correlations of liver inflammation grade with ALT (*P*=0.041, r=0.18), AST (*P*=0.001, r=0.29), and CHE (*P*=0.008, r=-0.23) were significant, while the correlations with HBsAg, ALB, and PLT were not significant. The correlation between liver fibrosis stage and age (*P* < 0.001, r = 0.32), TBIL (*P* = 0.008, r = 0.23), CHE (*P* < 0.001, r =-0.30), PLT (*P* < 0.001, r =-0.40), PTA (*P* = 0.046, r =-0.22) and INR (*P* = 0.04, r = 0.23) were significant, while the correlation with HBsAg, ALT, AST, and ALB were not significant. (**Table 3**)

**Table 3:** Histopathological grade distribution of the liver biopsy of HBeAg-negative and HBV DNA-negative patients.

Characteristics	G	S
	r/*P*	r/*P*
**age(years)**	0.07/0.417	0.32/<0.001
**HBsAg(log_10_ IU/ml)**	-0.12/0.28	-0.2/0.067
**AST(U/L)**	0.29/0.001	0.09/0.307
**ALT(U/L)**	0.18/0.041	0.08/0.382
**TBIL(μmol/L)**	0.12/0.159	0.23/0.008
**CHE(U/L)**	-0.23/0.008	-0.3/<0.001
**ALB(g/L)**	-0.16/0.076	-0.004/0.964
**PLT(10^9^/L)**	-0.12/0.16	-0.4/<0.001
**PTA(%)**	-0.09/0.451	-0.22/0.046
**INR**	-0.06/0.624	0.23/0.04

ALT, alanine aminotransferase; HBsAg, hepatitis B surface antigen; AST, aspartate aminotransferase; TBIL, total bilirubin; HBV DNA, hepatitis B virus deoxyribonucleic acid; ALB, albumin; INR, international normalized ratio; CHE, cholinesterase; PTA, prothrombin time activity; PLT, platelet.

### Logistic regression analysis

We performed logistic regression analysis on the differential indicators (age, TBIL, CHE and PLT) of two groups. The results of univariate logistic regression indicated that significant hepatic histopathology was associated with CHE level (OR = 0.018, 95% CI = 0.005 ~ 0.071, *P* < 0.001), age (OR = 1.077, 95% CI = 1.025 ~ 1.132, *P* = 0.003) and PLT count (OR = 0.988, 95% CI=0.985 ~ 0.992, *P* < 0.001). Multivariate logistic regression analysis indicated that only age (95% CI = 1.014 ~ 1.130, OR = 1.070, *P* = 0.015) was an independent factor. (**Table 4**)

**Table 4:** Logistic regression analysis of HBeAg-negative, HBV DNA-negative patients with and without significant hepatic histopathology.

Univariate binary logistic regression analysis				
Characteristics	B	*P*	OR	95% CI
**age(years)**	0.07	0.003	1.077	1.025~1.132
**TBIL(μmol/L)**	-0.96	0.059	1.051	0.998~1.106
**CHE(log_10_ U/L)**	-5.79	0.006	0.003	0.00005~0.190
**PLT(10^9^/L)**	-0.01	0.010	0.990	0.982~0.998
Multivariate binary logistic regression analysis				
** Characteristics**	**B**	***P***	**OR**	**95% CI**
** age(years)**	0.07	0.015	1.070	1.014~1.130
** TBIL(μmol/L)**	0.05	0.115	1.051	0.988~1.119
** CHE(log_10_ U/L)**	-0.0002	0.061	0.9997	0.9995~1.0001
** PLT(10^9^/L)**	-0.005	0.240	0.995	0.987~1.003
Cut off value and AUC				
** Characteristics**	**Cut off**	**sensitivity**	**specificity**	**AUC**
** age(years)**	34.30	78.90%	53.20%	0.688
** TBIL(μmol/L)**	14.65	55.30%	78.70%	0.643
** CHE(log_10_ U/L)**	8217.50	66.70%	71.10%	0.679
** PLT(10^9^/L)**	178.50	60.90%	70.30%	0.668

TBIL, total bilirubin; CHE, cholinesterase; PLT, platelet; AUC, area under curve.

**Figure 2:**
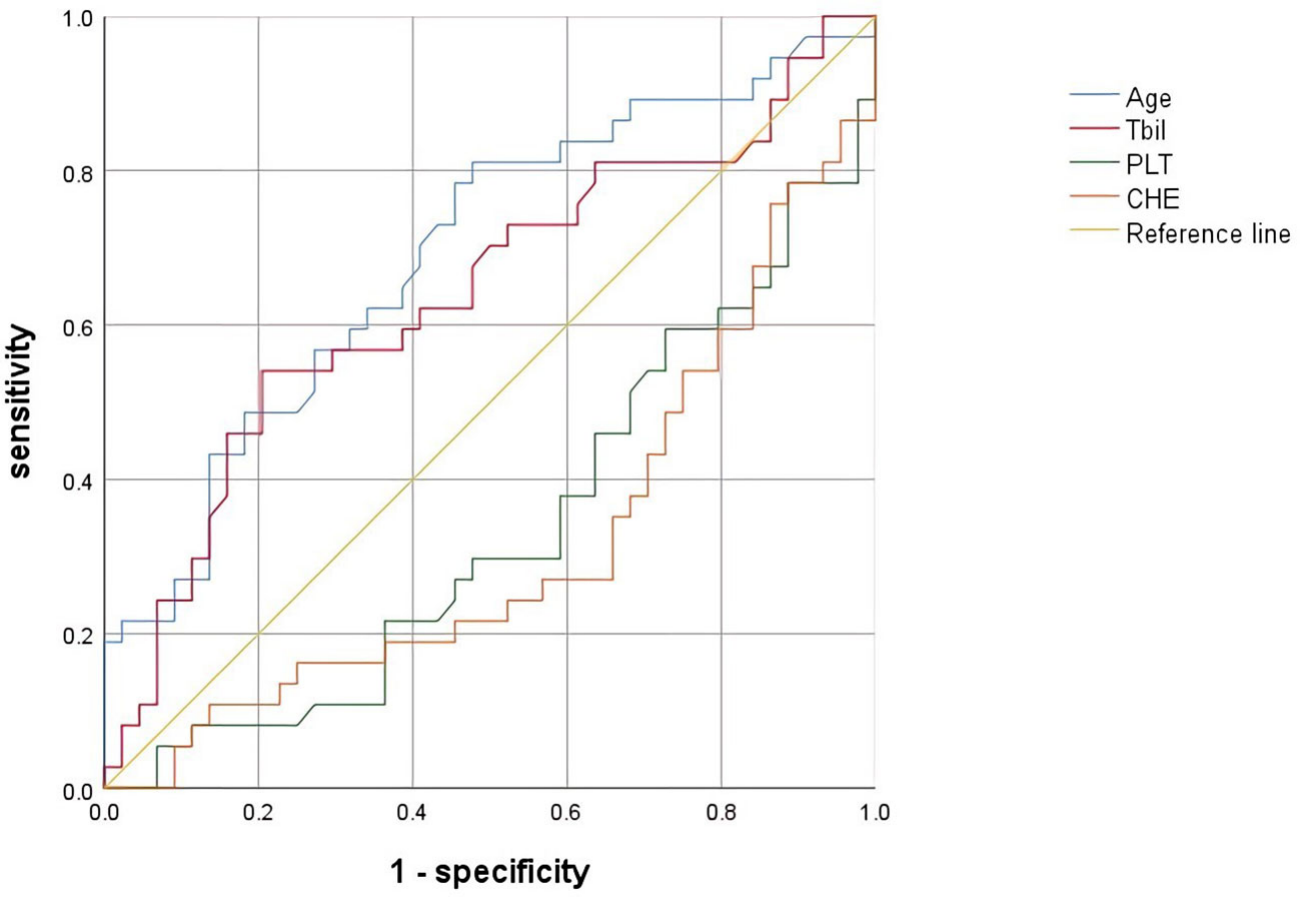
ROC of factors associated with significant hepatic histopathology. Abbreviations: CHE, cholinesterase; PLT, platelet; TBIL, total bilirubin; ROC, receiver operating characteristic curve.

The cutoff value was calculated by ROC curve and Jorden index, age 34.30 years, sensitivity 78.90%, specificity 53.20%, AUC=0.688. (**Table 4** and **Figure 2**)

## Discussion

During the natural history of chronic HBV infection, HBeAg seroconversion is a very important point, which implies a reduction or even silencing of viral cccDNA transcription activity (7). Also, in clinical practice, HBeAg seroconversion means a better prognosis because patients with HBeAg-negative chronic HBV infection are less likely to progress to cirrhosis or liver cancer. However, if the HBeAg-negative chronic hepatitis B period develops, there is still the possibility of disease progression (3, 8–10). That is to say, effective distinction between HBeAg-negative chronic hepatitis B and HBeAg-negative chronic HBV infection plays a very important role in determining the prognosis of those patients.

Martinot, Park H, Brunett prospectively included HBeAg-negative patients in their respective studies and investigated how to identify HBeAg-negative chronic hepatitis B patients from HBeAg-negative chronic HBV infection patients (11–13). Their results showed that HBsAg could be used as a factor in determining the presence of hepatitis in HBeAg-negative patients (11–13). Martinot and Oliveri, on the other hand, found that HBV DNA could also be used as a predictive factor in their own studies (11, 14). Brunetto found in his subsequent studies that HBcrAg was highly accurate in determining the presence of hepatitis in HBeAg-negative patients (15). However, the grouping methods in all these studies were by ALT and HBV DNA, yet in fact there are times when clinical indicators and liver pathology results do not parallel each other. Liver pathology results are the gold standard for determining the progression of disease. Therefore, we designed this study to investigate the risk factors for inflammation in HBsAg-positive, HBeAg-negative, and HBV DNA-negative patients using liver pathology findings as a grouping method and a more accurate HBV DNA assay.

Chronic hepatitis B is an immune-mediated disease, and age is an important factor in disease progression. Previous studies have shown a gradual decrease in the proportion of HBeAg-positive patients with increasing age, to less than 10% in patients over 40 years old (16). In our study, after comparing the clinical characteristics of significant hepatic histopathology group and no significant hepatic histopathology group, we found that patients with significant hepatic histopathology were older, and the correlation analysis showed that growing age was significantly related to the more severe fibrosis stage. Univariate and multivariate logistic regression showed that age was the independent impact factor of significant hepatic histopathology, meanwhile, the older the patient is, the more likely the patient has significant hepatic histopathology. Hsu Y S conducted a cohort study in HBeAg seroconversion patients and found that the annual incidence of hepatitis relapse was 2.2–3.3% (9). This study proved that with the age growing, more patients would develop hepatitis B. This is consistent with our findings. We calculated the cutoff value of age and it was 34.3 years old, and the AUC was 0.688, a barely satisfactory number.

CHE is an indicator of the synthetic function of the liver, which is affected when hepatitis or fibrosis occurs, and CHE decreases. This is confirmed by our study that patients with significant hepatic histopathology have a lower level of CHE, and with the growth of G and S, CHE decreases. Coagulation is also closely related to liver function, and our study found that PLT count is lower in patients with significant hepatic histopathology. PLT is negatively correlated to S according to the correlation analysis, PTA is also negatively correlated to S and INR is positively correlated to S. While we didn’t find similar results when it came to the correlation analysis with G. The findings indicated that liver fibrosis had a greater impact on Coagulation. ALT is a sensitive indicator of liver injury, but there are many causes of liver injury. Since we only included HBV DNA-negative patients for the study, for HBV DNA-negative patients, it is reasonable to consider more whether there are other causes of liver injury, so we did not consider ALT levels at the time of enrollment. In order to find out the reasons of liver injury, we conducted liver biopsy. In the comparison of the patients with and without significant hepatic histopathology, we did not observe a significant difference between ALT. But the comparison of normal ALT patients and abnormal ALT patients showed that there were more significant hepatic histopathology patients in abnormal ALT patients.

A study has shown that the incidence of hepatic flares ranges from 6% to 33% during the 2 to 7 years of follow-up (17), and intermittent flares may result in liver fibrosis. Therefore, pursuing a better endpoint, HBsAg seroclearance, is important. HBsAg seroclearance, also called “functional cure”, can effectively improve the long-term prognosis of patients and greatly reduce the risk of cirrhosis and liver cancer (18–20). Pegylated interferon α-2a (PEG-IFN α-2a) have a unique advantage over nucleoside (acid) analogues (NAs) in achieving HBsAg seroclearance (21–23).

Our study was a retrospective cross-sectional study. The enrolled patients were grouped by liver pathology results into two groups, significant hepatic histopathology group and no significant hepatic histopathology group. We studied the characteristics of clinical indicators and independent risk factors in HBsAg-positive, HBeAg-negative, HBV DNA-negative patients under high-sensitivity test assay. And we found that age was the independent risk factor. Our study has certain flaws, firstly the retrospective cross-sectional nature makes our findings need to be validated by a more scientifically sound prospective cohort study. Secondly, the volume of patients enrolled in our study was small. In the future, we may design a large-sample prospective cohort study to validate our findings and explore if there are more factors to recognize the presence of significant hepatic histopathology.

## Data availability statement

The original contributions presented in the study are included in the article/supplementary materials. Further inquiries can be directed to the corresponding author.

## Author contributions

ML and YX contributed to the study design. ZZ, RL, WC, YJL, LY and XB contributed to the data analysis. GS, MC, YL, SLW, MX, XC, LH, LZ and GW contributed to the recruitment, enrolment, and assessment of participants, as well as following up with the patients. TJ, WD, FS, HL and SHW contributed to data collection. ZZ wrote the first draft of the manuscript. YX and ML revised the manuscript and were the guarantor of the article. All authors contributed to the article and approved the submitted version.
